# Where to prospectively register a systematic review

**DOI:** 10.1186/s13643-021-01877-1

**Published:** 2022-01-08

**Authors:** Dawid Pieper, Tanja Rombey

**Affiliations:** 1 Faculty of Health Sciences Brandenburg, Brandenburg Medical School (Theodor Fontane), Institute for Health Services and Health System Research, Rüdersdorf, Germany; 2 Center for Health Services Research, Brandenburg Medical School (Theodor Fontane), Rüdersdorf, Germany; 3 Department of Health Care Management, Technische Universität Berlin, Berlin, Germany

**Keywords:** Systematic reviews, Meta-analyses, Registration, Registers, Transparency

## Abstract

**Background:**

Prospective registration aims to reduce bias in the conduct and reporting of research and to increase transparency. In addition, prospective registration of systematic reviews is argued to help preventing unintended duplication, thereby reducing research waste. PROSPERO was launched in 2011 as the first prospective register for systematic reviews. While it has long been the only option to prospectively register systematic reviews, recently there have been new developments. Our aim was to identify and characterize current options to prospectively register a systematic review to assist review authors in choosing a suitable register.

**Methods:**

To identify systematic review registers, we independently performed internet searches in January 2021 using keywords related to systematic reviews and prospective registration. “Registration” was defined as the process of entering information about a planned systematic review into a database before starting the systematic review process. We collected data on the characteristics of the identified registries and contacted the responsible party of each register for verification of the data related to their registry.

**Results:**

Overall, we identified five options to prospectively register a systematic review: PROSPERO, the Registry of Systematic Reviews/Meta-Analyses in Research Registry, and INPLASY, which are specific to systematic reviews, and the Open Science Framework Registries and protocols.io, which represent generic registers open to any study type. Detailed information on each register is presented in tables in the main text. Regarding the systematic-review-specific registries, authors have to trade-off between the costs of registration and the processing time of their registration record. All registers provide an option to search for systematic reviews already registered in the register. However, it is unclear how useful these search functions are.

**Conclusion:**

Authors can prospectively register their systematic review in five registries, which come with different characteristics and features. The research community should discuss fair and sustainable financing models for registers that are not operated by for-profit organizations.

## The purpose of prospective registration of systematic reviews

Prospective registration is a means to publish details about a research project before its commencement thus allowing evidence users to assess whether all steps of the research have been performed and reported as planned, or not. The overall aim is to reduce bias in the conduct and reporting of research and to increase transparency. The Declaration of Helsinki states that “[e]very research study involving human subjects must be registered in a publicly accessible database before recruitment of the first subject” [1]. In addition, prospective registration is legally required in the United States and Europe for some types of clinical trials [2, 3]. However, neither these laws nor the Declaration of Helsinki apply to the literature-based study type of systematic reviews. Nevertheless, one could argue that prospective registration is just as important for systematic reviews for they usually play an even greater role in evidence-based decision-making in clinical practice and health policy than single clinical trials [4]. In addition to reducing bias and increasing transparency, prospective registration of systematic reviews may prevent unintended duplication, thereby reducing research waste [5, 6].

Organizations conducting or commissioning systematic reviews related to health, such as Cochrane or the Joanna Briggs Institute (JBI), have their own databases of ongoing and published reviews. However, these databases are restricted to systematic reviews performed within these organizations. Thus, the idea of creating an independent registry of systematic reviews was proposed in January 2010 [7]. Following the establishment of a minimum dataset for prospective registration of systematic reviews [8], PROSPERO, the first international prospective register of systematic reviews, has been launched in February 2011. Nowadays, prospective registration of systematic reviews has been widely established and it is estimated that one third of systematic reviews published in 2018 were registered in PROSPERO [9].

From the viewpoint of authors, registers for systematic reviews need two main characteristics:

1. Offering the service to prospectively register a systematic review
2. Providing an oversight of all registered systematic reviews, including a search function

While the first one is obvious, the second one is particularly important when it comes to reducing unintended duplication of systematic reviews. All researchers intending to perform a systematic review should first search bibliographic databases and registers for systematic reviews on the same or similar research questions to avoid conducting duplicate reviews, which is already a major problem [10, 11].

Although PROSPERO has long been the only option to prospectively register systematic reviews, there recently have been new developments. Our aim was to identify and characterize current options to prospectively register a systematic review and thereby to assist review authors in choosing a suitable register.

## Scope

In this context, we define “prospective registration” as the action of entering information about a research project (here: a systematic review) into a database before its commencement. Usually, there is a pre-defined set of items the submitting authors are required to enter upon submission, such as the title, authors, aspects of the planned methods, and contact details. The depth of information is often left up to the authors, however. Prospective registration ought to be differentiated from publishing a manuscript for a protocol. Protocols are typically published as a stand-alone peer-reviewed article in a journal, as a registered report, or on a preprint server. The first two options have the advantage that the protocol manuscripts (unlike registration records and preprints) undergo peer-review which resembles a form of quality assurance. Furthermore, it can be assumed that journal publications have a high visibility as they are indexed in electronic databases.

We only considered registers for prospective registration of systematic reviews. Registers of completed systematic reviews, such as the Center on Knowledge Translation for Disability and Rehabilitation Research (KTDRR) Registry of Systematic Reviews [12], or KSR Evidence [13] are not further considered. Furthermore, we did not consider registers that are not universally open to all authors, such as the options offered by Cochrane, the Campbell Collaboration, the Agency for Healthcare Research and Quality (AHRQ), the JBI, or the Best Evidence Medical Education (BEME) Collaboration.

## Overview of current options to prospectively register a systematic review

To identify systematic review registers, the two authors independently performed internet searches in generic search engines (Google, DuckDuckGo) using keywords related to systematic reviews and prospective registration in January 2021. Furthermore, experts from the authors’ wider research network were contacted about their knowledge of further systematic review registers. We collected data on the registries identified in the previous step and organized the data in structured tables. Categories were developed deductively by the two authors. Regular meetings were held between the authors to agree on the categories. This process was informed by relevant literature, including several works of the authors. Following data collection, we contacted the responsible party of each register in April 2021 for verification of the data we had collected with regard to their registry.

Overall, we describe five options to prospectively register a systematic review: PROSPERO, the Registry of Systematic Reviews/Meta-Analyses in Research Registry, and INPLASY, which are specific to systematic reviews, and Open Science Framework (OSF) Registries and protocols.io, which present generic registers open to any study type. *OSF registries* specifically focuses on preregistration of studies, while the OSF in general is a project management tool for researchers with various functions, for example storing and publishing data. The key characteristics of these registers are reported in Table 1. Data have been verified for all registers except OSF Registries (no response received). At the end of the article, in addition to the five registries, other options are described for how and where to prospectively and transparently determine one’s planned methods.

**Table 1:** Characteristics of the identified registers

Register	Specific to systematic reviews			Generic	
	PROSPERO	Research Registry	INPLASY	OSF Registries	protocols.io
**Full name**	International prospective register of systematic reviews	Research Registry - Registry of Systematic Reviews/Meta-Analyses	International Platform of Registered Systematic Review and Meta-analysis Protocols	OSF Preregistration	protocols.io
**URL**	crd.york.ac.uk/prospero/	researchregistry.com/browse-the-registry#registryofsystematicreviewsmeta-analyses/	inplasy.com/	cos.io/initiatives/prereg	protocols.io
**Available since**	2011	2015	2020	2019	2014
**Costs**	None	99 GBP per systematic review	New registration: 20.90 USD or 12 USD (reduced price for selected countries). Update of registration: 8.90 USD (reduced price 4.90 USD).	None	None
**Number of registration records as per 27 February 2021**	> 100,000 (106,828)	> 1000 reviews (1051)	> 1000 (1446)	> 1000 (4152 when searching “systematic review”	> 100 (140 when searching “systematic review”)
**Assessment of records (e.g., for completeness, eligibility)**	Yes	Yes	Yes	No	No
**Processing time (submission to publication)**	No information	None (data curation takes place afterwards)	Max. 48 h	None	None
**Fields/structure**	28 mandatory fields, 12 optional fields	28 mandatory fields, 6 optional fields	24 mandatory fields, 9 optional fields	13 mandatory fields in OSF preregistration; 5 mandatory fields in Open-Ended-Registration	No given structure
**Enables searching**	Yes, text words, MeSH terms and many other characteristics	Yes, primary investigator, text words, title, and identifying number	Yes, text words and identifying number	Yes, text words only	Yes, text words only
**Provides contact address of corresponding author**	Yes	Yes	Yes	No	Not mandatory, but messages can be sent through protocols.io
**Reminders to keep the entry up to date?**	Yes	Unclear	No	No	Unclear
**Information on review status (i.e., completed/published)**	Yes	Link/Reference/DOI of full paper can be added once published	Yes	No	No
**Restricted to reviews?**	Yes, accepts only systematic reviews	Yes, accepts systematic reviews and meta-analyses	Yes, accepts systematic and scoping reviews	No, accepts all study designs	No, accepts all study designs
**Restricted to field/discipline?**	Health; but accepts also social care, welfare, public health, education, crime, justice, and international development if there is a direct health-related outcome	No	No	No	No
**Version tracking**	Yes	No	Yes	Yes	Yes
**Unique identifying number**	Yes	Yes	Yes	No	No
**DOI**	No	No	Yes	Yes	Yes
**Section for submitting results**	No	Yes, allows for uploading any data files	No	No, but can be linked to OSF project where results can be uploaded	No

PROSPERO seems to be the most prominent option by far, as indicated by their high number of registrations. However, this popularity also seems to have led to increased turn-around times. While there is no information on the overall processing time in PROSPERO, there is some evidence that it may take up to several months [14]. It can be assumed that in particular the assessment of submissions for eligibility and completeness take time. Such checks are also performed in Research Registry and INPLASY. However, registration records become immediately visible in Research Registry as submissions are assessed after registration, while INPLASY promises a fast turn-around time taking no longer than 48 hours. Although fast turn-around times are highly appreciated by authors [15, 16], they come at the price of a having to pay a fee for the services offered by Research Registry and INPLASY. This might be problematic for systematic reviews without specific funding. Submissions to OSF Registries or protocols.io are not formally assessed, but these registers are also free of charge. Another advantage of both is that they offer services to share the data collected as part of the systematic review.

The three registers specific to systematic reviews have a given structure consisting of 24–28 mandatory fields. In OSF Registries, users can select from different registration forms or chose “Open-Ended-Registration” and provide a narrative summary of their systematic review registration. All registers can be searched for systematic reviews already registered in the register. PROSPERO offers most search tools, including use of Medical Subject Headings. However, it has previously been reported that PROSPERO’s search function is suboptimal [17]. It is unclear how well the search functions of the remaining registers exactly work, but one could argue that more sophisticated search functions must be implemented to facilitate efficient searches as the number of registrations increases. In a perfect world, the search functions should be similar as in bibliographic databases. Authors’ contact details are provided in the three systematic-review-specific registers, but not in OSF Registries and protocols.io. PROSPERO, OSF Registries and protocols.io enable version tracking, and INPLASY, OSF Registries and protocols.io provide Digital Object Identifiers (DOIs) for registrations. Authors should note that there are differences between the registries with respect to their eligibility criteria. For example, PROSPERO only accepts reviews including at least one direct health-related outcome. Further information on the identified registers can be found in Table 2.

**Table 2:** Further information on the identified registers

Register	Specific to systematic reviews			Generic	
	PROSPERO	Research Registry	INPLASY	OSF Registries	protocols.io
**Launch references**	[18, 19]	[20, 21]	/	[22]	[23]
**Evaluations**	[24, 25]	[26, 27]	/	[28]	/
**Host**	Centre for Reviews and Dissemination (CRD) University of York York YO10 5DD United Kingdom	IJS Publishing Group 85 Great Portland Street Marylebone London W1W 7LT United Kingdom	INPLASY - International Platform of Registered Systematic Review and Meta-analysis Protocols Eugen-Huber-Strasse 12 8048 Zurich Switzerland	Center for Open Science 210 Ridge McIntire Road Suite 500 Charlottesville VA 22903-5083 United States	protocols.io 2120 University Ave Suite 625 Berkeley CA 94704 United States
**Founder(s)**	Alison Booth and Lesley Stewart (CRD) and Mike Clarke, Davina Ghersi, Gordon Dooley, David Moher, and Mark Petticrew (advisory group)	Riaz Agha	João Vitor Canellas	Brian Nosek, Jeffrey Spies (Center for Open Science)	Lenny Teytelman, Irina Makkaveeva, and Alexei Stoliartchouk
**Funding/business model**	Funded by United Kingdom National Institute for Health Research	For-profit organization	For-profit organization	Non-profit organization supported by grants	For-profit organization supported by grants and selling institutional licenses for private use

## Additional options to prospectively and transparently determine one’s planned methods

In addition to publishing a protocol and registering a systematic review in one of the five registers described above, we identified further options for prospectively reporting one’s planned methods. However, we do not consider them as registers (see definition above).

First, there are online open access data repositories allowing users to upload time-stamped, version-tracked files with a DOI. These services can be used to upload protocol documents, but also any other type of data associated with a research project. Examples include the OSF (open source; https://osf.io/), Figshare (commercial; https://figshare.com/), or Zenodo (open source;funded by CERN, OpenAIRE and the European Union; https://zenodo.org/). Using OSF and Zenodo is suggested as an option in the updated Preferred Reporting Items for Systematic reviews and Meta-Analyses (PRISMA) 2020 explanation and elaboration (Box 6) [29]. Recently, the PreregRS template for preregistering research syntheses was published, which aims to guide researchers in preparing a file that can be uploaded to such repositories [30]. More data repositories can be found by searching re3data.org, a registry of research data repositories. One option specifically for systematic reviews is the Systematic Review Data Repository by the AHRQ [31]. Second, we identified a pre-registration service called AsPredicted by the Penn Wharton Credibility Lab [32]. AsPredicted allows users to create a time-stamped PDF with the planned methods that can be shared selectively via a unique URL, but which remains private until an author makes it public. Third, we identified the Collaborative Approach to Meta-Analysis and Review of Animal Data from Experimental Studies (CAMARADES) by the Preclinical Systematic Review & Meta-analysis Facility (SyRF) [33]. SyRF shared protocols of systematic reviews of animal studies in in a standardized way on the CAMARADES website until 2018. Afterwards, the service was stopped, as systematic reviews of animal studies relevant to human health can nowadays be registered in PROSPERO. However, SyRF will continue to host systematic review protocols submitted previously [34]. Last, we also identified registration records for systematic reviews in ClinicalTrials.gov [35, 36]. While ClinicalTrials.gov aligns with our definition of a register, we did not consider it in our overview as it clearly has a different purpose, i.e., the prospective registration of clinical trials.

## Discussion

The five identified registers have different characteristics and features which authors may want to consider when choosing a suitable register for their next systematic review. It seems that, among the systematic-review-specific registries, authors have to trade-off between the costs of registration and the processing time. While being the costliest, Research Registry is also the fastest register as records are published immediately. PROSPERO is used widely and is free of charge, but as PROSPERO receives funding from the United Kingdom (UK) National Institute for Health Research (NIHR), registrations from the UK are prioritized. While this is understandable, it may be problematic for users from outside the UK. Furthermore, there is a potential risk that funding will be discontinued at some point as it has previously been the case with the National Health Service Economic Evaluation Database and the Database of Abstracts of Reviews of Effects that were also hosted by the CRD and funded through the UK NIHR [37]. Thus, it seems to be necessary to discuss alternative financing schemes for registries, which are both fair and sustainable. However, this is also so case for clinical trials registers. Originally, it has been discussed to include a systematic review registry in clinical trials registries to use existing technology and resources efficiently and promote collaboration among trialists and reviewers [7].

## Limitations

We identified several options to prospectively register a systematic review. While there are also important points regarding systematic review registers from a meta-perspective, this commentary is written from an author perspective. Furthermore, it is possible that we missed other available options and thus encourage others to share further options for the prospective registration of systematic reviews with us.

## Conclusion

We identified five registries where authors can prospectively register their systematic review, three of which are systematic-review-specific and two generic ones. These registries come with different characteristics and features, for example in terms of the costs of registration, turn-around times, and funding/business model. For registers that are not operated for-profit, e.g., PROSPERO, the research community should discuss fair and sustainable financing models to ensure long-term access and performance.

## Data Availability

All data generated or analyzed during this study are included in this published article.

## Abbreviations

AHRQ: Agency for Healthcare Research and Quality
BEME: Best Evidence Medical Education
CAMRADES: Collaborative Approach to Meta-Analysis and Review of Animal Data from Experimental Studies
CRD: Centre for Reviews and Dissemination
DOI: Digital Object Identifier
JBI: Joanna Briggs Institute
KTDRR: Center on Knowledge Translation for Disability and Rehabilitation Research
NIHR: National Institute for Health Research
OSF: Open Science Framework
PRISMA: Preferred Reporting Items for Systematic reviews and Meta-Analyses
SyRF: Systematic Review Facility
UK: United Kingdom
